# ELSMOR – towards European Licensing of Small Modular Reactors: Methodology recommendations for light-water small modular reactors safety assessment

**DOI:** 10.12688/openreseurope.16360.1

**Published:** 2023-09-21

**Authors:** Sylvain Lansou, Luca Ammirabile, Nikolai Bakouta, Jeremy Bittan, Sebastian Buchholz, Jean-Yves Brandelet, Etienne Courtin, Frans Davelaar, Stanislav Dombrovsky, Jean-Baptiste Droin, Sophie Ehster-Vignoud, Houda Hamama, Atte Helminen, Thorsten Hollands, Andriy Iskra, Sebastien Israel, Stefano Lorenzi, Liviusz Lovasz, Valerie Paulus, Isabelle Pichancourt, Joachim Miss, Thuy Nguyen, Antti Rantakaulio, Federico Rocchi, Juan-Carlos de-la-Rosa-Blul, Marco Ricotti, Armin Seubert, Oleksandr Sevbo, Stanislav Sholomitsky, Olli Suurnäkki, Marton Szogradi, Ville Tulkki, Andreas Wielenberg

**Affiliations:** 1 Framatome, Courbevoie, 92400, France; 2 Joint Research Center (JRC), European Commission, Brussels, 1049, Belgium; 3 Electricité de France (EDF), Paris, 75008, France; 4 Gesellschaft Fur Anlagen Und Reaktorsicherheit (GRS), Cologne, 50667, Germany; 5 Energorisk, Kiev, 03148, Ukraine; 6 Commissariat à l’Energie Atomique et aux Energies Alternatives (CEA), Paris, 75015, France; 7 VTT Technical Research Centre of Finland Ltd. (VTT), Espoo, 02150, Finland; 8 Institut De Radioprotection Et De Surete Nucleaire (IRSN), Fontenay aux Roses, 92260, France; 9 Consorzio Interuniversitario Nazionale per la Ricerca Tecnologica Nucleare (CIRTEN), Milano, 20156, Italy; 10 Fortum, Espoo, 02150, Finland; 11 Agenzia Nazionale Per Le Nuove Tecnologie, L’energia E Lo Sviluppo Económico Sostenibile (ENEA), Roma, 00196, Italy

**Keywords:** Small Modular Reactor, Safety, Reactivity Control, Decay Heat Removal, Confinement management, Severe Accident, Probabilistic Safety Assessment

## Abstract

Decarbonization of energy production is key in today’s societies and nuclear energy holds an essential place in this prospect. Besides heavy-duty electricity production, other industrial and communal needs could be served by integrating novel nuclear energy production systems, among which are low-power nuclear devices, like small modular reactors (SMRs). The ELSMOR (towards European Licensing of Small Modular Reactors) European project addresses this topic as an answer to the Horizon 2020 Euratom NFRP-2018-3 call.

The consortium includes 15 partners from eight European countries, involving research institutes, major European nuclear companies and technical support organizations. The 3.5-year project, launched in September 2019, investigates selected safety features of light-water (LW) SMRs with focus on licensing aspects.

Providing a comprehensive compliance framework that regulators can adopt and operate, the licensing process of such SMRs could be optimized, helping their deployment. In this prospect, as a result of ELSMOR’s work, this article gives an overview of the specific issues that LW-SMRs may bring about in the different domains of nuclear safety, in terms of:

• Methodological standpoints: safety goals, safety requirements, safety principles (defence-in-depth implementation);
• Main safety functions of reactivity control, decay heat removal and confinement management;
• Severe accident management;
• Other safety issues particular to SMRs: use of shared systems; performing of multi-unit probabilistic safety assessment (PSA); refuelling, spent fuel management, transport and disposal management.

In this article, adequate methodologies are developed to deal with these issues and to help assess the safety of LW-SMRs. This work gives a precious synthesis of the safety assessment issues of LW-SMRs and of the associated methodologies developed in the context of the ELSMOR European project.

## Plain language summary

The removal of fossil fuels in energy production is very important in today’s societies and nuclear energy plays an essential role in this. Besides large-scale electricity production, other industrial and communal needs could be solved by using new nuclear energy production systems, among which are low-power nuclear devices, like small modular reactors (SMRs). The ELSMOR (towards European Licensing of Small Modular Reactors) European project looks at this topic as an answer to the Horizon 2020 Euratom NFRP-2018-3 initiative.

This project includes 15 partners from eight European countries, involving research institutes, major European nuclear companies and technical support organizations. The 3.5-year project, started in September 2019, investigates selected safety features of light-water (LW) SMRs with a focus on the licensing aspects.

Providing a comprehensive compliance framework that regulators can use and operate, the licensing process of such SMRs could be optimized, helping their deployment. With this prospect, this article gives an overview of the specific subjects that LW-SMRs may bring in the different areas of nuclear safety (in particular: safety goals, safety requirements, nuclear safety functions: reactivity control, decay heat removal and confinement management, etc..).

In this article, methods are developed to deal with these new subjects and to help assess the safety of LW-SMRs. This work gives an overview of the safety assessment issues of LW-SMRs and of the associated methods developed in the context of the ELSMOR European project.

## Introduction

Decarbonization of energy production has become a central issue in today’s societies. Nuclear energy holds an essential place in this prospect. Besides heavy-duty electricity production, other industrial and communal needs could be served by integrating novel nuclear energy production systems, among which are low-power nuclear devices, like small modular reactors (SMRs).

The ELSMOR (towards European Licensing of Small Modular Reactors) project addresses this topic as an answer to the Horizon 2020 Euratom NFRP-2018-3 call (“ELSMOR Official Website”, http://www.elsmor.eu). The consortium includes 15 partners from eight European countries, involving research institutes, major European nuclear companies and technical support organizations. The 3.5-year project, launched in September 2019, investigates selected safety features of light-water (LW) SMRs with focus on licensing aspects.

SMRs promise a number of innovations in the domain of nuclear power. Such innovations may, for example, improve the speed of building and commissioning and the costs of the projects through the use of common and standardized designs, enabling series production. They may also bring technical benefits, such as increased autonomy and the possibility to extensively use passive safety features within the plant, which may be a safety asset.

In this context, one of the goals of ELSMOR goal is to create methods and tools for the European stakeholders to assess and verify the safety of LW-SMRs to be deployed in Europe. Providing a comprehensive compliance framework that regulators can adopt and operate, the licensing process of such SMRs could be optimized, helping their deployment.

## Project structure and progress

Activities have been thematized in 7+1 work packages (WPs), seven targeting different topics of SMRs and their specific safety features relevant for safety analyses, and one WP dedicated to project coordination. For demonstrative purposes, the main features of a new European SMR (E-SMR) design have been drafted in WP n°5.

This article will focus on the work performed in WP n°2. This work gives an overview of the specific issues that LW-SMRs may bring about in the different domains of nuclear safety, in terms of:

- Methodological standpoints: safety goals, safety requirements, safety principles (mainly defense-in-depth implementation, see: https://www.iaea.org/);
- Main safety functions of reactivity control, decay heat removal (DHR) and confinement management;
- Severe accident (SA) management;
- Other safety issues particular to SMRs: use of shared systems, performing of multi-unit probabilistic safety assessment (PSA), refueling, spent fuel management, transport and disposal.

Considering these safety domains, a set of safety requirements to be fulfilled by a LW-SMR in the prospect of its licensing in a European country was established. To tackle the identified safety issues, safety methodologies were studied or developed, and applied to case studies. Consequently, the results of WP2 should provide efficient tools for the licensing of the various LW-SMRs designs to be deployed in Europe.

A compilation of the safety conclusions made in this work is available (see: ELSMOR Deliverable 2.12: Synthesis: summary of methodology recommendations for LW-SMR safety assessment – S.Lansou, December 2021, available at: http://www.elsmor.eu/). In the present article, the main outcomes are presented.

## Methodology recommendations for LW-SMR safety assessment

### Recommendations related to high-level safety methodologies used for a licensing

SMRs’ innovative features may lead to adaptations in the way some safety principles and approaches (in particular Western European Nuclear Regulators' Association (WENRA) technologically neutral safety requirements) can be applied. The existing framework should still constitute the basis of the safety demonstration. Adequate methodologies should be developed to evaluate the application of the framework with respect to SMRs specificities. The main concerned subjects are: Defense-in-depth (DiD) implementation, severe accident, limitation of radiological consequences for accidents without and with core melt, resistance to hazards, autonomy of the plant and approaches for practical elimination of situations leading to large or early releases.

In this context, a set of high-level requirements to be fulfilled by LW-SMRs for their deployment in Europe was developed. These requirements are in line with international and European safety guidelines (International Atomic Energy Agency (IAEA), WENRA, European Nuclear Safety Regulators Group (ENSREG)). They account for SMR specificities, and they can help the designers to adopt specific design features limiting national specificities, thus spurring the licensing process. These requirements are presented in Table 1.

**Table 1.** High-level requirements to be fulfilled by light-water small modular reactors (LW-SMRs). *Acronyms used in the table – DiD: Defence-In-Depth, SMR: Small Modular Reactor, EPZ: Emergency Planning Zone, DBC: Design Basis Conditions, DEC-A: Design Extension Conditions without core melt, DEC-B: Design Extension Conditions with core melt*.

Domain	Requirement	Points of attention related to small modular reactors (SMR) designs
Defense-in-Depth (DiD)	DiD progressiveness and sufficient independence between DiD levels	Small modular reactors (SMR) tend to use passive systems. In such case, passive systems and SMRs safety characteristics are expected to provide alternative means to justify a sufficient independence between the different levels of DiD. In particular, such demonstration could rely on the combination of passive, active systems and SMR safety characteristics. However, these technologies address new challenges: • a limited operational experience; • uncertainties concerning their qualification and reliability assessments; • related operational aspects as periodic testing, maintenance and in-service inspections should be further studied.
	Forgiving DiD and grace time	Some SMR designs may give the opportunity to provide an enhanced forgiving defense thanks to a more favorable ratio between power and water inventory or broader operating margins. This must be justified.
	Emergency Planning Zone (EPZ)	SMRs features may contribute to a reduction of the size of the EPZ through the reinforcement of the safety demonstration and the resulting potential reduction of the radiological releases.
DBC (Design Basis Conditions)	List of DBCs	The design of SMRs may exclude some events (e.g., large breaks on primary loops for integrated SMRs). However, any exclusion should be drastically justified. Moreover, the introduction of new events challenging the plant safety functions by SMRs specificities should be accounted for.
DEC-A (Design Extension Conditions without core melt)	Types of DEC-A	Deterministic failure of SMR passive safety systems used for the limitation of DBC consequences should be considered as a DEC-A situation since they are not failure proof.
DEC-B (Design Extension Conditions with core melt)	Severe accident is postulated	The severe accident defined as the whole core melting accident must be considered and mitigated by DiD-level 4 measures. Indeed, despite scale and power reduction, the whole core melting accident remains physically possible if the fuel elements are not drastically modified (as compared to conventional cores). Excluding the whole core melting accident can only rely on physical impossibility.
	Independence of DEC-B safety features	For an SMR passive system, the claim of its high reliability cannot be enough to justify its use in all levels of DiD. To do so, only the physical impossibility of the function failure suffices.
Plant Autonomy	Autonomy of the electrical power supply and of the heat sink	This requirement must be verified considering the modularity of the plant. Conditions affecting several units or the fuel assembly storage pool (or both simultaneously) require a particular attention.
	Autonomy and external intervention	SMRs could be settled in remote regions, resulting in longer time for external resources to be provided. This should be accounted for in the safety demonstration.

In addition, requirements accounting for the plant modularity have been established. These requirements are presented in Table 2.

**Table 2.** Requirements accounting for the plant modularity. *Acronyms used in the figure: CCF: Common Cause Failure, DiD: Defence-In-Depth, DBC: Design Basis Conditions, DEC-A: Design Extension Conditions without core melt, DEC-B: Design Extension Conditions with core melt, SMR: Small Modular Reactor*.

Domain	Requirement	Points of attention related to small modular reactors (SMR) design
DBC (Design Basis Conditions)	Impact of an event on several units	The plant modularity implies the possibility, for an initiating event, to impact several units. This issue must be considered in DBC analysis.
DEC-A (Design Extension Conditions without core melt)	Multi-unit Common Cause Failure (CCF) from a common initiating event	It is required to treat as a DEC-A any CCF event impacting the DBC features of several units.
	Initiating multi-unit CCF	It is required to treat a CCF on independent safety normal operating systems of several units as a DEC-A.
Hazards	Propagation of hazards	Propagation of an internal hazard from a unit to another should be prevented. This should be accounted for in the safety demonstration.
	Extreme hazards on several units	The occurrence of an extreme external hazard (post-Fukushima situation) may impact all units of the plant. Large or early releases should be prevented by prevention and/or mitigation of fuel damage.
Multi-unit requirement	Systems shared among units	The safety demonstration should be provided for each unit, independently of others. Shared systems between units may impact the safety of the plant. The use of a system on a unit should not impair its capability to perform its safety function for other units when needed

**Figure 1.**
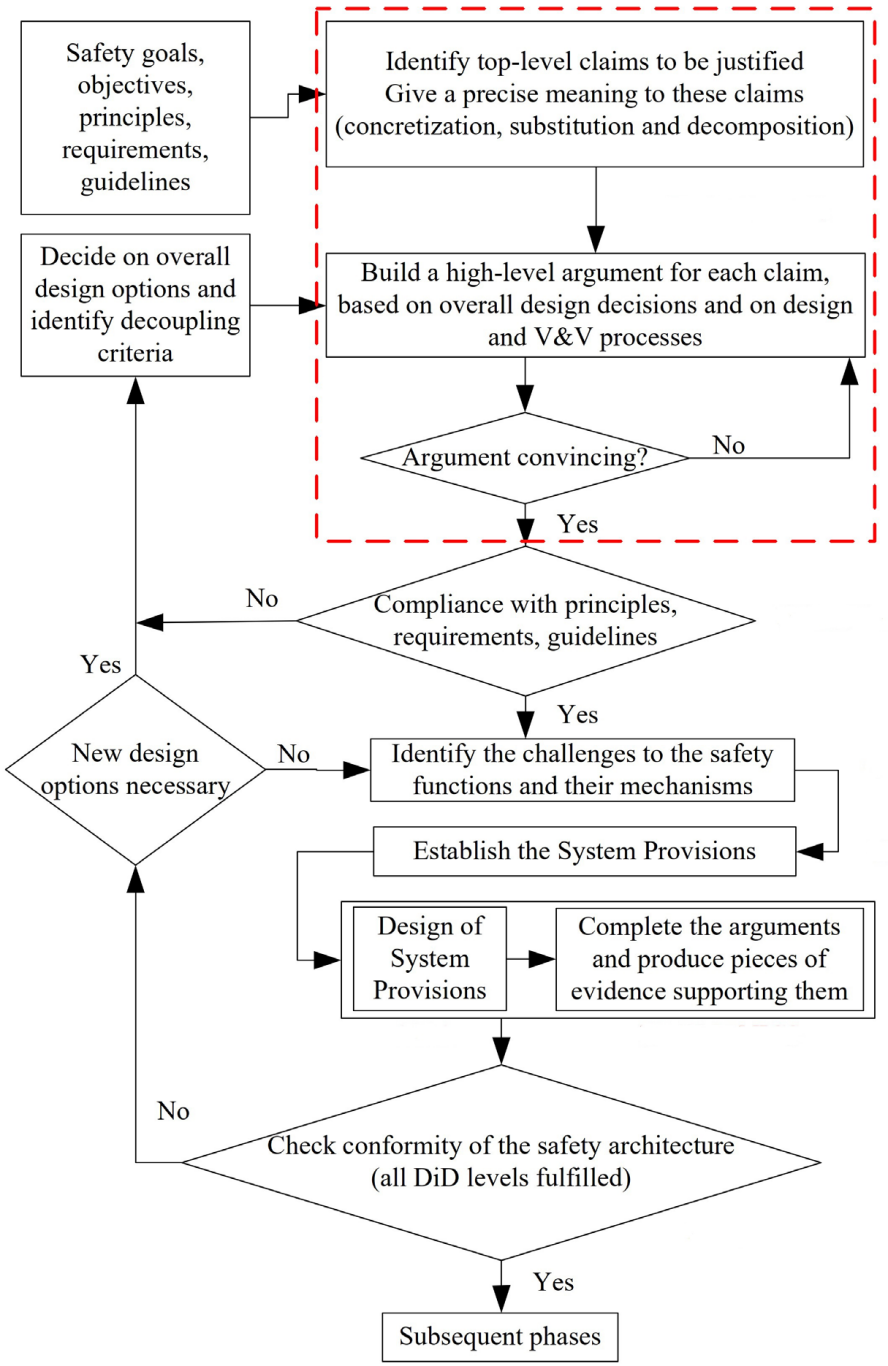
ELSMOR safety assessment methodology. (see ELSMOR Deliverable 2.2: Overview of safety methodologies for innovative reactor designs and proposal of a general methodology for LWSMR - L.Ammirabile, S.Buchholz, T. Nguyen, December 2020, available at: http://www.elsmor.eu/). *Acronyms used in the figure: DiD: Defence-In-Depth, V&V: Verification and Validation*.

In addition, a safety assessment methodology was developed to assess the safety of innovative reactors designs. It can contribute to the safety assessment of LW-SMRs. This methodology is accompanied by methods, appropriated from various methodologies (in particular, Integrated Safety Assessment Methodology (ISAM) developed by the Gen IV International Forum's Risk and Safety Working Group (GIF RSWG)). The complete algorithm is illustrated in Figure 1.

As part of this general ELSMOR’s methodology, a flexible, non-accident specific methodology, called Graphical Independence of DiD assessment (GID), has been developed for subsequent safety demonstration. GID may be used during conceptual design phases. The method provides the functions and sub-functions that have to be enabled to ensure the fundamental safety functions in all plant states. This allows the checking of independence between the main systems involved in the performance of these functions at various DiD levels. In this way, GID was applied to the heat removal function of the E-SMR (WP5) in power operation.

### Recommendations related to the implementation of the main safety functions by the LW-SMR

In the context of the safety assessment of a nuclear reactor, three fundamental safety functions should be controlled for the reactor, for all its plant states (power operation, hot shutdown, cold shutdown, including refueling operations) and for all levels of DiD: the control of the reactivity, the heat removal and the confinement of radioactive materials. The safety requirements related to these functions in various countries were studied and their applicability to LW-SMRs was verified.

***Reactivity control.*** In large, pressurized water reactors (PWR), safety criteria related to reactivity control are satisfied by inherent fuel characteristics, by the control rods and the boric acid injection system in the primary water.

For SMRs, the reactivity control should also rely on the same inherent fuel characteristics and mainly on control rods (CR), particularly for boron-free designs (this design decision may be driven by economics and depends on the plant power level). Thus, the control of the geometry of the fuel assemblies is essential: distortion, bowing, damages due to mechanical forces/stress should be considered. Criticality events due to maintenance issues are also of concern in this context.

Uncertainties associated with the design of SMRs CRs should be assessed. It is needed to justify the CR insertion in case they are credited, in particular in loss of coolant accident (LOCA) scenarios, as LOCAs can induce distortion of the core barrel and misalignment of CRs.

The CRs insertion rate by gravity drop in case of emergency shutdown (SCRAM) could be an issue, because of the limited height of the core and the limited weight of the CRs. This could lead to a decreased insertion speed compared to large PWRs. The speed of insertion by gravity drop should therefore be carefully evaluated.

Concerning the redundancy and diversity of shutdown systems, IAEA SSR-2/1 states that “ *The means for shutting down the reactor shall consist of at least two diverse and independent systems.*” and that “ *At least one of the two different shutdown systems shall be capable, on its own, of maintaining the reactor subcritical by an adequate margin and with high reliability, even for the most reactive conditions of the reactor core.*” ^1^. In this regard, SMR designs tend to rely mainly on CRs as the first shutdown system and implement as a second one the fast injection of boric acid into the primary system by either active or passive driving forces. Considering the borication system, special attention should be paid to the long-term stability of the correct boron concentration.

For some SMRs, the cancelation of the use of boron (completely or partially) for the control of the reactivity in normal operation is proposed. For SMRs relying on burnable neutron poisons (rather than on a borication system), IAEA’s SSG-52 ^2^ requests the evaluation of the effects of a depletion of burnable absorbers on the core reactivity to ensure an adequate shutdown margin in all resulting applicable core conditions throughout the operating cycle. Such designs have several advantages from a safety aspect. The elimination of certain accident scenarios is possible (boron dilution). The operational flexibility is improved (no dilution time during operation, simplification of the maintenance, reduction of effluent wastes). The radioprotection is improved as well (reduction of up to 1/3 of the tritium production). However, certain safety issues have to be tackled in the safety demonstration: the disappearance of a redundant reactivity control system and the increased reliance on CRs (necessary to increase the effectiveness of the CRs: use of particular absorbing materials, an increase of the number of CR pins or of CRs). Consequently, there may be a potential need to exclude rod ejection by the use of innovative solutions. A faster depletion of CRs due to their increased exposure to neutrons may require more frequent inspections. The degree of reliability of innovative systems will have to be proved, experimental data being necessary. Moreover, attention should be paid to some potential situations in which the shutdown margin would not be sufficient to prevent some recriticality after shutdown in the long term (i.e. in cold shutdown).

***Decay heat removal (DHR).*** The following recommendations have been established to ensure a robust safety demonstration of the DHR function.

The reliability of passive DHR systems has to be demonstrated. Methodologies devoted to their reliability assessment have been developed in the frame of European R&D activities and of EURATOM projects (see 3 and 4). All types of failures should be considered in safety DHR systems (single, passive, functional and common cause failure (CCF)). In particular, specific failure modes for passive safety systems are identified in the reliability assessment methodologies. They refer mainly to thermohydraulic failures, leading to functional failures. For example, a degradation of heat transfer capabilities or pressure drops in the heat exchanger tube bundle, may lead to fluid dynamic instability and oscillating/reduced flowrate, inducing a functional degradation in terms of released thermal power to the heat sink. These passive systems may be subject to spurious actuation and this should be accounted for (this is not related to their passive character).

Passive decay heat removal systems (DHRS) are subject to a two-phase flow thermohydraulic operation. Hence the thermohydraulic codes used for their simulation need to be qualified for several related phenomena, in particular: natural circulation in the passive loop and in the water pool, subcooled nucleate, saturated boiling or condensation on the tube or plate walls (in-tube, ex-tube, within plates) of the heat exchangers and effects of non-condensable gases.

***Confinement.*** The following recommendations have been established to ensure a robust safety demonstration of the confinement function.

Passive heat removal through containment wall

For NUWARD ^TM^-type designs, the containment vessel is submerged in a large water pool. In some accident conditions, inflowing steam is condensed on the containment inner wall and heat is transferred into the pool. Consequently, the only element available to control the pressure inside the containment during a LOCA is the condensation on the containment wall and the resulting heat transfer to the pool.

The containment integrity must be ensured, despite overpressure, under-pressure or thermal loads.

It must be demonstrated that for all kinds of accidents, in particular LOCA and main steam line breaks (MSLB) the heat transfer into the large water pool is sufficient to keep containment integrity. This includes the effect of non-condensable gases on the condensation heat transfer. The more compact containment design of SMRs comes with the potential for more severe and possibly faster overpressure transients; however, the exclusion of a large break from the design reduces short-term loads.

Impacts of earthquakes

The impact of large pools on the resistance of the plant toward earthquakes is an issue. Seismic waves will induce oscillations of the water pool outside the containment. At the same time, seismic waves will be transferred to the containment vessel via its connection to the ground plate, which may be in phase with pool oscillations. A detailed analysis should expose occurrence of peak loads to containment structures that may challenge its leak-tightness. On the pool side, it should be demonstrated that there is no unacceptable loss of pool inventory for containment cooling due to earthquakes so that the pool remains available as a heat sink in case of design basis conditions (DBC) and design extension condition (DEC) scenarios.

Wetwell/pool

Certain designs (e.g., Flexblue, CAREM) present a containment composed of several separate compartments (wetwell and drywell). In this case, transport processes of non-condensable gases can lead to their accumulation in specific compartments, affecting local temperatures and pressures. This can pose specific challenges for the operation of passive heat removal systems and induce heat and pressure loads to the containment.

### Issues related to severe accident management

Extreme physical conditions such as the one the facility may encounter during a severe accident (SA) should be considered in the safety assessment. The aim is to verify that the facility can perform its functions despite such extreme conditions. It is expected that the low power of SMRs does not question the major principles established for the safety demonstration regarding SA management for large PWR. SMR-related issues are highlighted in the following paragraph.

Objectives should be defined in terms of potential impacts between units and on populations in case several units are involved. Referring to SA scenarios, an ELSMOR emergency planning zone (EPZ) assessment methodology was developed for SMR multi-unit plants (see 5). For the E-SMR, the full-scope determination of EPZ distances for the E-SMR will be performed using the output data of the DEC analyses in WP5.

SMRs extensively rely on passive systems for the prevention and mitigation of core melting (e.g., passive in-vessel corium retention by ex-vessel cooling, external flooding of the reactor pressure vessel (RPV) from water tanks, etc.). In this regard, the difficulty of assessing the reliability of passive devices, particularly in the context of design extension conditions with core melt (DEC-B) scenarios (extreme and widely varying conditions) has been highlighted. Consequently, a set of requirements on the credit of passive systems for DEC-B scenarios has been established. These requirements mainly concern the need for:

•     A demonstrated reliability of the passive systems in extreme DEC-B conditions. These systems should be designed for boundary conditions including high or extreme pressures and temperature fields. These systems must be demonstrated to reliably achieve their missions over the full range of conditions they are likely to experience with robust demonstration. It should be demonstrated that there are no cliff-edges near the mission envelope and adequate safety or margins should be achieved by design.

•     The ability of operators to deal with these systems: severe accident dedicated safety provisions must have the necessary instrumentation to get the essential information to the operators, limiting the missing information about the system status or abilities. This instrumentation should be designed according to the DEC-B physical conditions and support operators with suitable human machine interfaces (HMI).

To the extent possible, passive systems used in DEC-B should be tested under realistic severe accident conditions.

The use of innovative equipment for SMRs and their associated issues regarding DEC-B sequences management has to be considered (integrated design, compact containment, alternative cladding and fuel material, boron-free coolant).

The limits of the tools and codes used in Europe for SA calculations are of concern, especially regarding particular phenomena: debris beds formation, cooling, melting, crust formations, steel relocation paths, etc.

SMRs tend to adopt a severe accident management strategy based on in-vessel melt retention (IVMR). In this regard, safety requirements were established:

•     Molten corium retention: the realistic thickness of the metallic layer on a corium pool in the lower plenum should be known and its limited impact on the RPV be demonstrated.

•     Reactor pit flooding: to realize IVMR strategy, the outer RPV wall has to be flooded with water. Sufficient water sources in the containment and the RPV have to be ensured to do so.

•     Heat removal: Effective heat removal by natural circulation and recirculation into the RPV are claimed. The effectiveness of these systems must be ensured in case of SA. Sufficient liquid level in the reactor pit must guarantee natural circulation. Any risk of steam blockage, due to higher local heat flux, and limiting the wall cooling should be avoided.

•     Ultimate heat sink (UHS): to ensure the long-term feasibility of IVMR strategy. It is necessary to demonstrate the long-term availability of the UHS.

### Safety issues particular to SMRs

Some safety issues, which are SMR-related were studied. They consist of the potential multi-unit character of the SMR plant and the management of systems shared among SMR units.

***Shared systems.*** An extensive use of equipment shared between the units is foreseen for SMRs: auxiliary systems (e.g., boron supply, demineralized water supply), control rooms, pools used as UHS, etc. This may raise issues:

- Initiating events affecting several units of the plant simultaneously may occur (LOOP, failure of a steam line in case of a common turbine…);
- An initiating event should not induce hazardous effects on neighboring units;
- Some shared equipment can be used to mitigate consequences of accidents occurring simultaneously in several units.

In line with the previous points, IAEA TECDOC 1936 ^6^ mentions that each unit of a multi-modular facility should dispose of its own safety systems for design-extension conditions, when possible. If a safety system or safety device is shared between several units, the shared safety system or safety device must be functionally capable of meeting the safety requirements of each unit or of all units simultaneously.

***Multi-unit PSA.*** One key characteristic of SMRs is their installation within a multi-unit plant. This impacts PSA quantifications.

The traditional risk matrix should be extended to incorporate multi-unit sequences. As one of the major factors in PSA, CCF quantification has to be revisited, particularly considering the number of impacted equipment (which may be too high on a multi-unit site to provide an accurate quantification) and nature (whether the equipment is involved on several units or not). These modifications of the risk matrix would also justify the need for new probabilistic numerical targets (i.e., event frequency).

***Refueling, spent fuel management and disposal.*** It has been shown that the approaches and techniques used to justify the safety of large-power reactors are applicable to SMRs. A smaller size of fuel assemblies and a smaller mass of fuel, with a similar level of burnup, would reduce the severity of the consequences of major design accidents. Yet, some design features of SMRs induce issues regarding accidents related to the spent fuel pool (SFP) of the plant, as illustrated below.

For SMR boron-free designs, an important amount of gadolinium may be used in the fuel as a burnable neutron poison. This may increase the importance of the gadolinium peak in the fuels compared to PWR. This may be an issue during outages phases of partially spent fuel. This phenomenon should be considered in the design of SFP racks.

The use of innovative equipment and passive systems for SMRs also induces particular requirements for fuel management:

•     Practical elimination of the SFP fuel damage accidents must consider SMR specificities (reactivity aspects: clear water in the SFP, fuel reactivity; use of passive systems; plant modularity …);

•     Any potential for severe consequences to arise (consideration of worst conditions for common SFP with full cores stored from all reactors) should be identified.

•     If passive heat removal systems are used for the SFP (single-phase heat removal system, for example), their performance must be demonstrated (proven codes, adequate modeling of related physical phenomena, experimental support).

Moreover, the mutual impacts between the SFP and the reactors should be considered in case the fuel is stored within the containment (as in Water-Water Energy Reactor (VVER) technologies for example):

•     SMR modules and the SFP can be impacted through their shared systems (e.g., supply of cooling water to SFP cooling system and to diesel generators, power supply, ventilation).

•     An accident in an SMR module can impact the SFP.

•     An accident in the SFP can impact an SMR module: flooding due to SFP system piping rupture (if applicable), accidents in the SFP leading to conditions which require emergency shutdown of the unit by the personnel.

•     Management of accident sequences should consider both the reactors and the SFP.

Considering decommissioning, onsite decommissioning for multi-unit plants may be sequenced (decommissioning of units while some others are still operating). This would require particular safety resolutions (implementation of particular removal routes, works close to operating units...).

## Synopsis and outlook

ELSMOR tackles an array of critical aspects of light-water SMR licensing. The project establishes an assessment methodology for such purposes, based on extensive experimental and analytical work. The work performed in WP2 has permitted an overview of the different issues that LW-SMRs may bring about in the different domains of nuclear safety, in terms of:

•     Methodological standpoints (safety goals, safety requirements);

•     Main safety functions of reactivity control, decay heat removal and confinement management;

•     Severe accident management and Emergency Planning Zones (EPZ);

•     Safety issues peculiar to SMRs: shared systems, multi-unit PSA aspects and refueling, spent fuel management, transport and disposal.

The upcoming European TANDEM project (“TANDEM Official Website”, http://tandemproject.eu/) will study the use of SMR designs for subsequent cogeneration plant studies, considering H2 production, district heating and power supply for urban areas. In this prospect, the safety analysis methodologies developed in ELSMOR may be used and adapted.

In addition, the safety methodologies developed through this work may be used in the prospect of the licensing of the various LW-SMRs designs to be installed in Europe. It may ease the licensing processes as it provides a set of safety requirements to be fulfilled by a LW-SMR in the prospect of licensing in Europe and developed various methodologies to tackle a set of identified safety issues.

## Ethics and consent

Ethical approval and consent were not required.

## Data Availability

No data are associated with this article.
