# Supplementary material for: ELSMOR – towards European Licensing of Small Modular Reactors: Methodology recommendations for light-water small modular reactors safety assessment
Source: Open Res Eur. 2024 Oct 14;3:158. Originally published 2023 Sep 21. [Version 2] doi: 10.12688/openreseurope.16360.2 (PMC11487226; doi:10.12688/openreseurope.16360.2)
Supplement: Peer review history

# Reviewer report: Reviewer response for version 2

**DOI:** 10.21956/openreseurope.20133.r45132

**Published:** 2024-10-22

**Authors:** Edward Shitsi

**Affiliations:** 1 National Nuclear Research Institute, Ghana Atomic Energy Commission, University of Ghana, Kwabenya,, Accra, Ghana

All the Reviewer comments have been addressed satisfactorily.

Where applicable, are recommendations and next steps explained clearly for others to follow? (Please consider whether others in the research community would be able to implement guidelines or recommendations and/or constructively engage in the debate)

Yes

Does the article adequately reference differing views and opinions?

Partly

Are all factual statements correct, and are statements and arguments made adequately supported by citations?

Yes

Is the rationale for the Open Letter provided in sufficient detail? (Please consider whether existing challenges in the field are outlined clearly and whether the purpose of the letter is explained)

Yes

Is the Open Letter written in accessible language? (Please consider whether all subject-specific terms, concepts and abbreviations are explained)

Yes

Reviewer Expertise:

Reactor safety and thermal-hydraulics

I confirm that I have read this submission and believe that I have an appropriate level of expertise to confirm that it is of an acceptable scientific standard.

# Reviewer report: Reviewer response for version 2

**DOI:** 10.21956/openreseurope.20133.r45131

**Published:** 2024-10-17

**Authors:** Andrej Prosek

**Affiliations:** 1 Jožef Stefan Institute - Ljubljana, Ljubljana, Slovenia

With the exception of two typos,  the authors in REVISED version considered given comments.

Where applicable, are recommendations and next steps explained clearly for others to follow? (Please consider whether others in the research community would be able to implement guidelines or recommendations and/or constructively engage in the debate)

Partly

Does the article adequately reference differing views and opinions?

Partly

Are all factual statements correct, and are statements and arguments made adequately supported by citations?

Yes

Is the rationale for the Open Letter provided in sufficient detail? (Please consider whether existing challenges in the field are outlined clearly and whether the purpose of the letter is explained)

Yes

Is the Open Letter written in accessible language? (Please consider whether all subject-specific terms, concepts and abbreviations are explained)

Yes

Reviewer Expertise:

nuclear safety, nuclear safety requirements, deterministic safety analyses

I confirm that I have read this submission and believe that I have an appropriate level of expertise to confirm that it is of an acceptable scientific standard.

# Reviewer report: Reviewer response for version 1

**DOI:** 10.21956/openreseurope.17661.r38164

**Published:** 2024-08-14

**Authors:** Edward Shitsi

**Affiliations:** 1 National Nuclear Research Institute, Ghana Atomic Energy Commission, University of Ghana, Kwabenya,, Accra, Ghana

This project/work investigates selected safety features of light-water (LW) SMRs with focus on licensing aspects and methodology recommendations. The work gives an overview of the specific subjects relating to licensing of LW-SMRs that need to be considered in different areas of nuclear safety (in particular: safety goals, safety requirements, safety principles (defense-in-depth implementation); nuclear safety functions (reactivity control, decay heat removal and confinement management); severe accident management; and other safety issues (use of shared systems; performing of multi-unit probabilistic safety assessment (PSA); refueling, spent fuel management, transport and disposal management)).

 This work is publishable to help guide regulators and operators to address the necessary safety concerns regarding deployment of LW-SMRs in a commercial scale. But the following issues need to be addressed before indexing:

1. All the sub-titles should be logically numbered reflecting the main titles.
2. Other safety issues relating to licensing that have not been considered in this work should also be mentioned in the “Synopsis and outlook” or in a new subtitle “Scope”, else there must be statement clearly stating that all the necessary safety requirements relating to licensing have been considered.
3. The successful deployment of NPPs (nuclear power plants) in a commercial scale depends largely on the design and Operation related activities aside regulatory supervision activities. In terms of licensing, the methodology recommendations for LW-SMR safety assessment provided, are these recommendations covering all the safety issues relating to design and operation of LW-SMRs?
4. The methodological recommendations provided for LW-SMR safety assessment is based on the methodology shown in figure 1 (ELSMOR safety assessment methodology). Are these recommendations applicable to any other methodology for SMR safety assessment? Provide the basis for “Yes or No” answer.
5. Consider the subtitle “Refueling, spent fuel management and disposal”, the write-up under this subtitle contains no information on “refueling”. The authors should include some information on “refueling” or otherwise, modify the subtitle.
6. The authors might have read different licensing requirements required for licensing NPPs before coming out with this article. The authors should mention some of these requirements reviewed in writing this article. 

Where applicable, are recommendations and next steps explained clearly for others to follow? (Please consider whether others in the research community would be able to implement guidelines or recommendations and/or constructively engage in the debate)

Yes

Does the article adequately reference differing views and opinions?

Partly

Are all factual statements correct, and are statements and arguments made adequately supported by citations?

Yes

Is the rationale for the Open Letter provided in sufficient detail? (Please consider whether existing challenges in the field are outlined clearly and whether the purpose of the letter is explained)

Yes

Is the Open Letter written in accessible language? (Please consider whether all subject-specific terms, concepts and abbreviations are explained)

Yes

Reviewer Expertise:

Reactor safety and thermal-hydraulics

I confirm that I have read this submission and believe that I have an appropriate level of expertise to confirm that it is of an acceptable scientific standard, however I have significant reservations, as outlined above.

# Response

**Published:** 2024-09-23

**Authors:** Sylvain Lansou

**Affiliations:** Framatome, France

Dear Edward Shitsi, Thank you for your review. The following updates have been made to integrate your remarks:

1. Numbers were added to each title
2. The objective of the work performed in the task 2.1 of the ELSMOR project was to propose a set of requirements for LW-SMRs related to the defence-in-depth, to the Design basis conditions, to the Design extension conditions without core melt, to the Design extension conditions with core melt, to the practical elimination of situations, to plant autonomy, to hazards and to multi-modules, for which adequate safety methodologies must be developed to evaluate whether the safety objectives are fulfilled or not. As such, for these requirements, the aim of the project was to cover the most structuring safety domains of nuclear safety. Such statement will be added to the article.
3. The work performed in the WorkPackage number 2 of the ELSMOR project are preliminary and do not cover all the licensing issues for an NPP, choices of subjects were sometimes done with a focus on LW-SMRs specific thematics related to SMR design (passive systems, modularity of the plant, etc.)  and operation (common control room for several modules for example, studied in deliverable 2.9). Security and impact studies were not investigated in the frame of our WorkPackage number 2 work for example.
4. Most of the requirements described in the article are applicable to other LW-SMR projects, even if different safety methodologies may have been used.
5. The term “refueling” was deleted.
6. Table 1 and 2 will be updated to integrate references to the regulations considered with cross-links towards reference bibliographic documents. A paragraph called “State-of-the-art of ongoing LW-SMRs safety assessment and of ongoing LW-SMR concepts will be added in the beginning of the document. It will describe the work done in terms of Nuclear safety directives and good practices on safety assessment of LW-SMRs reviewed. This review was based on information from: European safety directives, IAEA guidelines, WENRA (Western European Nuclear Regulators' Association) guidance, ENSREG (European Nuclear Safety Regulators Group) guidance, National rules and regulations on selected EU and non-EU (Canada, Russia, USA) countries that are currently in an SMR licensing process. A state of the art of ongoing SMR projects will be added as well. Tables 1 and 2 will be completed with references, for each requirement provided, to higher goal requirements taken from the licensing literature analyzed by the ELSMOR project.

  We sincerely hope our comments can answer your observations and we thank you again for your review. Best regards,

# Reviewer report: Reviewer response for version 1

**DOI:** 10.21956/openreseurope.17661.r38626

**Published:** 2024-08-14

**Authors:** Fatih Aydogan

**Affiliations:** 1 Jacksonville University, Jacksonville, Florida, USA

Thanks to all the authors for submitting this article.

 1. The citations and references should be expanded and clarified. For instance, IAEA's main website is cited for the methodology standpoints, etc. Specific websites and references will be useful for the community and readers. 

 2. Is there a typo for "WP n°5" since it is not clear what "°" is? Needs updates or clarification.

 3. SMRs are FOAK products so that there is no experience of SMRs.  Thus, "a limited operational experience" term may be updated.

 4. "The design of SMRs may exclude some events (e.g., large breaks on primary loops for integrated SMRs) can be revised since the main idea of the SMRs is eliminate LB-LOCAs by using their unique design features. 

 5. Based on the passive and inherent safety features of SMRs, currently there is no severe accident risk. If DiD- Level-4 has a scenario to use some of the reactor or power cycle components, various scenarios can be mentioned that should or can be considered. 

 6. The statement of "The plant modularity implies the possibility, for an initiating event, to impact several units." should be clarified since modularity is used for different engineering designs: 1. for multiple units such as NuScale units, 2. Modular manufacturing and construction of SMR parts, such as W-SMR parts. In Table 2, the term "modularity" used for SMRs like NuScale is criticized.  Therefore, the statements about modularity need to be clarified.

 7. Analytic and probabilistic safety analyses may be mentioned in Figure.1 since a quantitative method is needed for licensing an SMR.

Where applicable, are recommendations and next steps explained clearly for others to follow? (Please consider whether others in the research community would be able to implement guidelines or recommendations and/or constructively engage in the debate)

Yes

Does the article adequately reference differing views and opinions?

Partly

Are all factual statements correct, and are statements and arguments made adequately supported by citations?

Partly

Is the rationale for the Open Letter provided in sufficient detail? (Please consider whether existing challenges in the field are outlined clearly and whether the purpose of the letter is explained)

Yes

Is the Open Letter written in accessible language? (Please consider whether all subject-specific terms, concepts and abbreviations are explained)

Yes

Reviewer Expertise:

SMRs, Power/Enegy Systems, Artificial Intelligence, Design, Computer Simulations, R&D

I confirm that I have read this submission and believe that I have an appropriate level of expertise to confirm that it is of an acceptable scientific standard, however I have significant reservations, as outlined above.

# Response

**Published:** 2024-09-23

**Authors:** Sylvain Lansou

**Affiliations:** Framatome, France

Dear Fatih Aydogan, Thank you for your review. The following updates have been made to integrate your remarks:

1. Additional references were added. A paragraph called “State-of-the-art of ongoing LW-SMRs safety assessment and of ongoing LW-SMR concepts will be added in the beginning of the document. It will describe the work done by ELSMOR in terms of Nuclear safety directives and good practices on safety assessment of LW-SMRs reviewed. This review was based on information from: European safety directives, IAEA guidelines, WENRA (Western European Nuclear Regulators' Association) guidance, ENSREG (European Nuclear Safety Regulators Group) guidance, National rules and regulations on selected EU and non-EU (Canada, Russia, USA) countries that are currently in an SMR licensing process. A state of the art of ongoing SMR projects will be added as well. Tables 1 and 2 will be completed with references, for each requirement provided, to higher goal requirements taken from the licensing literature analyzed by the ELSMOR project.
2. Modified: changed to “WP number 5”. This abbreviation will not be used anymore in the text.
3. Changed to “no or limited operational experience”. The term “limited” is kept as for some components, a certain level of operational experience may already exist: plate type heat exchanger for instance, which are already used in some non-nuclear concepts (chemical industry for example).
4. The sentence is reformulated into the following: “Some events are excluded in the design of SMRs”
5. From the ELSMOR work, it is addressed that the severe accident shall be deterministically considered in the prospect of the implementation of DiD according to WENRA recommendations. Severe accident is considered in the design of SMRs in Europe as part of the deterministic approach, which remain the base of the safety demonstration. The probabilistic approach only complements it but may not suffice to permit the non-consideration of a severe accident in the design of the LW-SMR. Thus, the possibility for a severe accident to occur on a LW-SMR must be considered, despite the intrinsic “interesting” safety features of the SMR. Despite the passive character of a system, there is a level of passivity that may vary depending on the human actions or the automatic “active” actions that may be necessary to actuate or keep in operation the system. Even if a passive system may be considered as “fully passive”, its efficient operation will involve active systems (associated to ventilation or heat sink for example). The failure of these active systems, that may encompass the ability of the passive system considered to achieve its function must be considered, as successive failures may lead to a severe accident.
6. The modularity term will be clarified in the paragraph “Recommendations related to high-level safety methodologies used for a licensing”
7. The Figure was changed for a more complete one, which includes, in particular, references to PSA methods.

  We sincerely hope our comments can answer your observations and we thank you again for your review. Best regards,

# Reviewer report: Reviewer response for version 1

**DOI:** 10.21956/openreseurope.17661.r38167

**Published:** 2024-03-06

**Authors:** Andrej Prosek

**Affiliations:** 1 Jožef Stefan Institute - Ljubljana, Ljubljana, Slovenia

This Open Letter focused on the work performed in Work package 2 of H2020 Euratom ELSMOR project, where ELSMOR project Work Package 2 focuses on developing methodologies with recommendations to support the safety demonstration of the innovative features of Light Water-Small Modular Reactors (LW-SMRs).

 It seems that this article summarizes the deliverable D2.12 entitled "Synthesis: summary of methodology recommendations for LW-SMR safety assessment", which is a complete summary of the work realized in the context of Work Package 2 tasks. It gives an overview of the specific issues that LW-SMRs may bring about in the different domains of nuclear safety, in terms of methodological standpoits, main safety functions, severe accident management and other safety issues particulars to SMRs.

 The Open Letter provides useful information, with the note that not all results obtained the frame of ELSMOR project are presented. For example, for the set of high-level requirements to be fulfilled by LW-SMRs for their deployment in Europe, only those with specificities for SMRs are presented, with reference to D2.12. As D2.12 deliverable is again summary, the reader has further to refer to reports D2.1 through D2.11. In this respect a table listing all identified requirements (from generic to multi-modules, around 35 in total) would be useful to the readers or brief information, how many of identified requirements in the frame of ELSMOR project are specific for SMRs (this would eliminated the need to dig further into ELSMOR deliverables).

 It is recommended also to consider the following specific comments:

 1. Check wording in 'Introduction': Use of "goals" and "goal" not clear in the sentence "In this context, one of the **goals** of ELSMOR **goal** is …".

 2. Between initial and name space is suggested, see last paragraph of section 'Project structure and progress': "S.Lansou".

 3. Table 2, last row, last column: Period is missing at the end of last sentence.

 4. Figure 1 is simplified comparing to Figure 1 of ELSMOR deliverable D2.2, which identifies also the steps of the ELSMOR methodology and the review methodologies (e.g. HARMONICS, QSR, PIRT, GID). The Open Letter has section 'Recommendations related to high-level safety methodologies used for a licensing', but later very few review methods are mentioned (e.g. ISAM is exception). Consider to provide some information or at least references for the review methodologies.

 5. For reference 2 the title of IAEA SSG-52 specific safety guide is suggested to be added.

 6. Section 'Recommendations related to the implementation of the main safety functions by the LW-SMR', 'Decay heat removal (DHR)': Check text "considered in safety DHR systems" (expected "considered in DHR safety systems").

 7. Section 'Recommendations related to the implementation of the main safety functions by the LW-SMR', 'Decay heat removal (DHR)': With respect to paragraph "Passive decay heat removal systems (DHRS) are subject to a two-phase flow thermohydraulic operation. Hence the thermohydraulic codes used for their simulation need to be qualified for several related phenomena…" it is suggested to consider the following WENRA document:

 Regulatory Aspects of Passive Systems, https://www.wenra.eu/sites/default/files/publications/rhwg_passive_systems_2018-06-01_final.pdf

 With respect to the thermohydraulic codes used for simulations it is not enough just to qualify the thermohydraulic codes, but to properly consider input data. WENRA RHWG Report on Regulatory Aspects of Passive Systems under O3.2 states that for safety assessment care has to be taken to the specific range of conditions necessary to perform the safety function, taking into account that this range of conditions could be narrow. Namely, specific range of conditions (in particular when associated with uncertainties) may have consequences on safety analysis of safety function performance.

 8. Section 'Recommendations related to the implementation of the main safety functions by the LW-SMR', 'Confinement', 'Impacts of earthquakes': Check last sentence " On the pool side, … and design extension condition (DEC) scenarios."

 Abbreviation DEC is typically used for "Design Extension Conditions" (not "Design Extension Condition").

 9. Section 'Issues related to severe accident management', second paragraph: Check wording "For the E-SMR, the full-scope determination of EPZ distances for the E-SMR will be performed using the output data of the DEC analyses in WP5." The future tense is used, while the ELSMOR project is finished.

 10. Section 'Safety issues particular to SMRs', 'Shared systems', last paragraph: check wording "design-extension" (in original IAEA TECDC-1936 wording "design extension" is used).

Where applicable, are recommendations and next steps explained clearly for others to follow? (Please consider whether others in the research community would be able to implement guidelines or recommendations and/or constructively engage in the debate)

Partly

Does the article adequately reference differing views and opinions?

Partly

Are all factual statements correct, and are statements and arguments made adequately supported by citations?

Yes

Is the rationale for the Open Letter provided in sufficient detail? (Please consider whether existing challenges in the field are outlined clearly and whether the purpose of the letter is explained)

Yes

Is the Open Letter written in accessible language? (Please consider whether all subject-specific terms, concepts and abbreviations are explained)

Yes

Reviewer Expertise:

nuclear safety, nuclear safety requirements, deterministic safety analyses

I confirm that I have read this submission and believe that I have an appropriate level of expertise to confirm that it is of an acceptable scientific standard, however I have significant reservations, as outlined above.

# Response

**Published:** 2024-09-23

**Authors:** Sylvain Lansou

**Affiliations:** Framatome, France

Dear Andhika Yudha Prawira,

 Thank you for your review.

 The following updates have been made to integrate your remarks:

 A precision in the description of table 1 and table 2 was added to emphasize that table 1 concerns single-unit requirements and table 2 focuses on multi-unit requirements.

 The requirements described in these tables are the ones to which we estimated the reader should point his attention at.

 However, to access to your request, a sentence to describe the fact that further requirements were developed in the project, in particular in ELSMOR Deliverable 2.1 (public deliverable, accessible here: LW-SMRs main safety goals - N. Playez, E. Courtin, L. Ammirabile, S. Israel ( https://www.elsmor.eu/wp-content/uploads/2021/12/ELSMOR_D2_1__final_signed.pdf)  will be added with a link towards the deliverable, where all requirements are described exhaustively. Concerning other reviewer remarks:

1. Corrected
2. Corrected
3. Corrected
4. A description of the methodologies reviewed (ISAM, INPRO, SARGEN_IV, HARMONICS) and used for the development of the ELSMOR safety assessment methodology has been added in the text of the article. References of the reviewed methodologies and the description of their content are described in ELSMOR Deliverable 2.2 to which a cross-link has been added in the text.

Moreover, apart from the development of this methodology, a paragraph called “State-of-the-art of ongoing LW-SMRs safety assessment and of ongoing LW-SMR concepts will be added in the beginning of the document. It will describe the work done in terms of Nuclear safety directives and good practices on safety assessment of LW-SMRs reviewed. This review was based on information from: European safety directives, IAEA guidelines, WENRA (Western European Nuclear Regulators' Association) guidance, ENSREG (European Nuclear Safety Regulators Group) guidance, National rules and regulations on selected EU and non-EU (Canada, Russia, USA) countries that are currently in an SMR licensing process. A state of the art of ongoing SMR projects will be added as well.

 In addition, Table 1 and 2 will be updated to integrate references to the regulations considered. A sentence to describe the fact that further requirements were developed in the project, in particular in Deliverable 2.1 will be added with a link towards the deliverable, where all requirements are described exhaustively.

1. Title added
2. Corrected
3. The following sentence will be added in the paragraph: “Input data must be properly considered as the range of conditions necessary to perform the safety function could be narrow for passive systems, especially when associated with uncertainties in the model correlations, in the initial conditions and in the boundary conditions (see [5], available at: https://www.wenra.eu/sites/default/files/publications/rhwg_passive_systems_2018-06-01_final.pdf).”
4. Corrected
5. Corrected (“was” used)
6. Corrected

  We sincerely hope our comments can answer your observations and we thank you again for your review. Best regards

# Reviewer report: Reviewer response for version 1

**DOI:** 10.21956/openreseurope.17661.r37797

**Published:** 2024-02-26

**Authors:** Andhika Yudha Prawira

**Affiliations:** 1 Nuclear and Quantum Engineering, Korea Advanced Institute of Science and Technology, Daejeon, Daejeon, South Korea

The manuscript describes the result of the ELSMOR (towards European Licensing of Small Modular Reactors) European project, which provides a comprehensive methodology to address Light Water Small Modular Reactor (LW-SMR) regulatory compliance. The ELSMOR goal is to create tools and methods to assess and verify the safety of LW-SMR for the stakeholders in Europe.

1. The article provides explanations of the existing regulatory requirements. However, I think it will be better to include some references to the referred regulation/TECDOC/requirements in Table 1. It will better represent differing views/approaches needed for LW-SMR and help address the potential points of exclusion and/or added requirements. 
2. Sentences related to the Control Rods (CR) need to be revisited, especially concerning the "limited height of the core and the limited weight of CRs." A new table can be added to better represent the difference in safety requirements in the case of CRs requirement with a location system (Boron concentration requirement) and Boron-free design (using burnable neutron poisons). 
3. A new section and/or table can be added to better visualize the difference between single-unit and multi-unit SMR requirements. The impact of shared systems and the need for multi-unit PSA can be more justified when clear and concise distinctions are provided between single-unit and multi-unit deployment.
4. All in all, the manuscript is a good open letter and can contribute to the field of SMR safety licensing. Improvements can be made by providing more information on the necessary modifications to the current safety requirements and highlighting the unique characteristics of SMR design. The manuscript may provide examples with citations on the ongoing state-of-the-art SMR project in which safety goals, features, and requirements may be better assessed using ELSMOR methodologies.

Where applicable, are recommendations and next steps explained clearly for others to follow? (Please consider whether others in the research community would be able to implement guidelines or recommendations and/or constructively engage in the debate)

Partly

Does the article adequately reference differing views and opinions?

Partly

Are all factual statements correct, and are statements and arguments made adequately supported by citations?

Yes

Is the rationale for the Open Letter provided in sufficient detail? (Please consider whether existing challenges in the field are outlined clearly and whether the purpose of the letter is explained)

Yes

Is the Open Letter written in accessible language? (Please consider whether all subject-specific terms, concepts and abbreviations are explained)

Yes

Reviewer Expertise:

SMR safety, security, and safeguard (3S), nuclear security, nuclear energy policy.

I confirm that I have read this submission and believe that I have an appropriate level of expertise to confirm that it is of an acceptable scientific standard, however I have significant reservations, as outlined above.

# Response

**Published:** 2024-09-23

**Authors:** Sylvain Lansou

**Affiliations:** Framatome, France

Dear Andhika Yudha Prawira, Thank you for your review. The following updates have been made to consider your remarks:

1. A paragraph called “State-of-the-art of ongoing LW-SMRs safety assessment and of ongoing LW-SMR concepts will be added in the beginning of the document. It will describe the work done by ELSMOR in terms of Nuclear safety directives and good practices on safety assessment of LW-SMRs reviewed. This review was based on information from: European safety directives, IAEA guidelines, WENRA (Western European Nuclear Regulators' Association) guidance, ENSREG (European Nuclear Safety Regulators Group) guidance, National rules and regulations on selected EU and non-EU (Canada, Russia, USA) countries that are currently in an SMR licensing process. A state of the art of ongoing SMR projects will be added as well. In addition, Tables 1 and 2 will be updated to integrate references to the regulations considered by the ELSMOR project to issue the safety requirements. A sentence to describe the fact that further requirements were developed in the project, in particular in ELSMOR Deliverable 2.1 (public deliverable), will be added as well with a link towards the deliverable, where all requirements are described exhaustively.
2. The sentence highlighted by your comment on control rods will be completed and will be changed into the following one:

“For SMRs, the CRs insertion rate by gravity drop in case of SCRAM could be an issue, because of two factors: the limited height of the core and the limited weight of the CRs themselves. These two factors, influencing non-linearly the mechanical friction resistance, the fluid resistance, and the fluid-solid coupling, resulting in the interaction between gravity, buoyancy, and friction, might lead to an overall decreased speed of insertion with respect to that of large PWRs. The speed of insertion by gravity drop should therefore be carefully evaluated both in the design phase and in the safety assessment for licensing.” It is believed, from the ELSMOR work, that “Referring to the insertion of the CR, taking a footprint similar to a CR, it is not influenced by the mass of the CR itself, but mostly by the density difference between the CR and water.” is a good preliminary approach to initiate a design. However, at a later design stage, to have a clear vision of the correct insertion times, complementary evaluations and experiments would be needed. The precise insertion speed is difficult to calculate as it is the result of several non-linear phenomena. However, it is believed from the ELSMOR work that Safety Authorities, will require experimental data, not only calculations, to approve the design of the control rods. In the J04-6 paper “Analytical Modeling of Control Rod Drop Behavior, M.Ren and J.Stabel, Transactions of the 15 ^th^ International Conference on Structural Mechanics in Reactor Technonology (SMiRT-15) – Seoul, Korea, August 15-20,199”, the relation between drop speed and core height is shown for a KONVOI PWR (experiments vs calculations) in Figure 5.

1. Table 2 concerns multi-unit requirements and Table 1 concerns single-unit requirements. Titles will be changed to display this more clearly. A reference to table 2 will be added in the “Safety issues particular to SMRs” part.
2. In addition to the actions taken and described in the first bullet point of this text, complementary sentences were added in the conclusion to highlight the fact that the ongoing SMR projects may benefit from the ELSMOR work.

  We sincerely hope our comments can answer your observations and we thank you again for your review. Best regards,

# Reviewer report: Reviewer response for version 1

**DOI:** 10.21956/openreseurope.17661.r35238

**Published:** 2023-11-02

**Authors:** Fabio GIANNETTI

**Affiliations:** 1 Sapienza University of Rome, Roma, Italy

The manuscript deals with the work done in the H2020 ELSMOR project WP2, with a focus on the possible specific issue of the LW-SMRs.

 The quality of the manuscript is enough for publication as an open letter. The only improvement needed is the insertion of a clear state-of-the-art SMR safety assessment.

 A specific concern needs to be addressed regarding the sentence: "The CRs insertion rate by gravity drop in case of emergency shutdown (SCRAM) could be an issue, because of the limited height of the core and the limited weight of the CRs. This could lead to a decreased insertion speed compared to large PWRs. The speed of insertion by gravity drop should therefore be carefully evaluated."

 Referring to the insertion of the CR, taking a footprint similar to a CR, it is not influenced by the mass of the CR itself, but mostly by the density difference between the CR and water.

Where applicable, are recommendations and next steps explained clearly for others to follow? (Please consider whether others in the research community would be able to implement guidelines or recommendations and/or constructively engage in the debate)

Yes

Does the article adequately reference differing views and opinions?

Partly

Are all factual statements correct, and are statements and arguments made adequately supported by citations?

Yes

Is the rationale for the Open Letter provided in sufficient detail? (Please consider whether existing challenges in the field are outlined clearly and whether the purpose of the letter is explained)

Yes

Is the Open Letter written in accessible language? (Please consider whether all subject-specific terms, concepts and abbreviations are explained)

Yes

Reviewer Expertise:

Nuclear thermal-hydraulics, GEN IV and SMR safety assessment

I confirm that I have read this submission and believe that I have an appropriate level of expertise to confirm that it is of an acceptable scientific standard, however I have significant reservations, as outlined above.

# Response

**Published:** 2024-09-23

**Authors:** Sylvain Lansou

**Affiliations:** Framatome, France

Dear Fabio GIANNETTI, Thank you for your review. The following updates have been made to consider your remarks:

- A paragraph called “State-of-the-art of ongoing LW-SMRs safety assessment and of ongoing LW-SMR concepts will be added in the beginning of the document. It will describe the work done by ELSMOR in terms of Nuclear safety directives and good practices on safety assessment of LW-SMRs reviewed. This review was based on information from: European safety directives, IAEA guidelines, WENRA (Western European Nuclear Regulators' Association) guidance, ENSREG (European Nuclear Safety Regulators Group) guidance, National rules and regulations on selected EU and non-EU (Canada, Russia, USA) countries that are currently in an SMR licensing process. A state of the art of ongoing SMR projects will be added as well.

 

- The sentence highlighted by your comment on control rods will be completed and will be changed into the following one:

“For SMRs, the CRs insertion rate by gravity drop in case of SCRAM could be an issue, because of two factors: the limited height of the core and the limited weight of the CRs themselves. These two factors, influencing non-linearly the mechanical friction resistance, the fluid resistance, and the fluid-solid coupling, resulting in the interaction between gravity, buoyancy, and friction, might lead to an overall decreased speed of insertion with respect to that of large PWRs. The speed of insertion by gravity drop should therefore be carefully evaluated both in the design phase and in the safety assessment for licensing.” It is believed, from the ELSMOR work, that “Referring to the insertion of the CR, taking a footprint similar to a CR, it is not influenced by the mass of the CR itself, but mostly by the density difference between the CR and water.” is a good preliminary approach to initiate a design. However, at a later design stage, to have a clear vision of the correct insertion times, complementary evaluations and experiments would be needed. The precise insertion speed is difficult to calculate as it is the result of several non-linear phenomena. However, it is believed from the ELSMOR work that Safety Authorities, will require experimental data, not simply calculations, to approve the design of the control rods. In the J04-6 paper “Analytical Modeling of Control Rod Drop Behavior, M.Ren and J.Stabel, Transactions of the 15 ^th^ International Conference on Structural Mechanics in Reactor Technonology (SMiRT-15) – Seoul, Korea, August 15-20,199”, the relation between drop speed and core height is shown for a KONVOI PWR (experiments vs calculations) in Figure 5.

 We sincerely hope our comments can answer your observations and we thank you again for your review.

 Best regards,
